# Plastic and Reconstructive Surgeons' Knowledge and Comfort of Contralateral Prophylactic Mastectomy: A Survey of the American Society of Plastic Surgeons

**DOI:** 10.3389/fonc.2018.00647

**Published:** 2019-01-09

**Authors:** Christopher D. Lopez, Rachel Bluebond-Langner, Carrie A. Houssock, Sheri S. Slezak, Emily Bellavance

**Affiliations:** ^1^Icahn School of Medicine at Mount Sinai, New York, NY, United States; ^2^Wyss Department of Plastic Surgery, NYU Langone Health, New York, NY, United States; ^3^Department of Plastic and Reconstructive Surgery, Johns Hopkins Hospital, Baltimore, MD, United States; ^4^Department of Surgery, University of Maryland School of Medicine Baltimore, Baltimore, MD, United States

**Keywords:** contralateral prophylactic mastectomy, surgical decision making, breast reconstruction, contralateral breast cancer risk, oncologic benefit

## Abstract

**Background:** Despite limited oncologic benefit, contralateral prophylactic mastectomy (CPM) rates have increased in the United States over the past 15 years. CPM is often accompanied by breast reconstruction, thereby requiring an interdisciplinary approach between breast and plastic surgeons. Despite this, little is known about plastic surgeons' (PS) perspectives of CPM. The purpose of this study was to assess PS practice patterns, knowledge of CPM oncologic benefits, and perceptions of the CPM decision-making process.

**Methods:** An electronic survey was sent to 2,642 members of the American Society of Plastic Surgeons (ASPS). Questions assessed demographics, practice patterns, knowledge of CPM oncologic benefits, and perceptions of the CPM decision-making process.

**Results:** ASPS response rate was 12.5% (*n* = 329). Most responders worked in private practice (69%), were male (81%) and had been in practice for ≥15 years (60%). The median number of CPM reconstructions performed per month was 2–4. Fifty-five percent of PS reported routine attendance at a breast multidisciplinary conference. Responders reported CPM discussion was most likely to be initiated by the patient (51%) followed by the breast surgeon (38%), and plastic surgeon (7.3%). According to PS, the most common reason patients choose CPM is a perceived increased contralateral cancer risk (86%). Most plastic surgeons (63%) assessed the benefits of CPM as worth the risk of additional surgery and the majority (53%) estimated the complication rate at 2X the risk of unilateral surgery. The majority (61%) of PS estimated risk of contralateral cancer in an average risk patient between <2 and 5% over 10 years, which is consistent with data reported from the current literature. Most plastic surgeons (87%) reported that there was no evidence or limited evidence for breast cancer specific survival benefit with CPM. A minority of PS (18.5%) reported discomfort with a patient's choice for CPM. Of those surgeons reporting discomfort, the most common reasons for their reservations were a concern with the risk/benefit ratio of CPM and with lack of patient understanding of expected outcomes. Common reasons for PS comfort with CPM were a respect for autonomy and non-oncologic benefits of CPM.

**Discussion:** To our knowledge, this is the first survey reporting PS perspectives on CPM. According to PS, CPM dialogue appears to be patient driven and dominated by a perceived increased risk of contralateral cancer. Few PS reported discomfort with CPM. While many PS acknowledge both the limited oncologic benefit of CPM and the increased risk of complications, the majority have the opinion that the benefits of CPM are worth the additional risk. This apparent contradiction may be due to an appreciation of the non-oncologic benefits CPM and a desire to respect patients' choices for treatment.

## Background

Despite evidence supporting the use of breast conservation in the treatment of operable breast cancer (1–4), contralateral prophylactic mastectomy (CPM) procedures have been on the rise (5–12). Several national databases have observed a large increase in the number of CPM in early stage breast cancer treatment (6, 8).

Women who undergo CPM report increased confidence with their decision and less worry about cancer recurrence, suggesting that fear could in fact be a potential impetus for the rise of CPM (7). The incidence of bilateral mastectomy is higher in women with greater income (6, 13) and private insurance (14), suggesting that access to care could also be contributory. However, the ability to access the “peace of mind” that CPM offers fearful patients is an incomplete answer, since this trend in the United States is absent in Europe (12).

Greater access to the reconstructive surgery (14, 15), and a unique cultural acceptance of plastic surgery in the United States (12) may also play a role. Breast reconstruction has been shown to be an independent predictor of CPM (16–18). The close relationship between CPM and reconstruction highlights the need to elucidate the role of the plastic surgeon (PS) in CPM decision making.

The purpose of this survey was to assess the PS perspective and knowledge of CPM in order to help define their role in the decision-making process of CPM. In addition, given the limited oncologic benefit of CPM and increased perioperative complication rate of an additional mastectomy with reconstruction, we also assessed plastics surgeons' comfort with patients' requests for CPM.

## Methods

This study was approved by the University of Maryland School of Medicine Institutional Review Board. A self-administered 15-question electronic survey was sent to members of the American Society of Plastic Surgeons (ASPS). Questions were selected for inclusion in this survey were based on the senior author's experience in developing a similar tool for oncologic surgeons. In short, the questions developed were based on a literature review and interdisciplinary clinical expertise from breast cancer surgeons, plastic surgeons, ethicists, and others (19). The survey was approved and distributed by the ASPS. There was no financial incentive to complete the survey.

Survey questions assessed demographic information, practice patterns, reasons to initiate a discussion about CPM, perceptions of why patients choose CPM, and surgeon knowledge of oncologic benefit of CPM. Surgeons were asked if they had ever felt uncomfortable with a patient's choice for CPM and asked to articulate reasons for discomfort in an open-ended format (Appendix 1). All survey data were coded and entered into a database. Descriptive statistics are reported as proportions. Analysis of the data was performed using SAS statistical software (SAS version 9.3; SAS Institute Cary, NC). Statistical significance of observed differences was calculated using Chi Square tests. A *P* ≤ 0.05 was considered significant. Qualitative analysis was used to analyze the open-ended responses. Familiarization of open-ended responses was obtained with repeated reading of the text by 3 independent reviewers. Recurrent themes were identified and an index of major themes was applied to the data by annotating each response. Data were charted by extracting the text of the responses and were arranged according to thematic reference.

## Results

### Demographics and Practice Patterns

**Figure 1:**
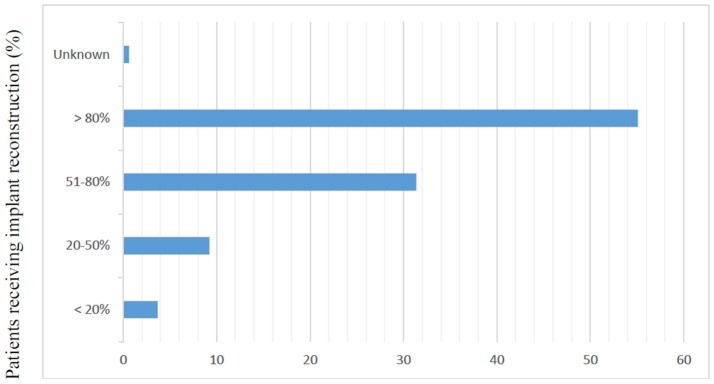
Responses to question, “In your practice, what proportion of patients receive implant based reconstruction?” (*N* = 325).

Of the 2,642 members of ASPS who were sent the questionnaire, 329 (12.5%) responded. Most responders (81%) were male and worked in private practice (68.9%). Slightly more than half of responders (54.8%) reported routine attendance of multidisciplinary breast conference and were in practice for at least 10 years (77%). Only 6.7% of surgeons polled reported over 80% of their cases are breast reconstruction. The median reported number of CPM reconstructions performed per month was 2–4 (Table 1). The majority of responders performed implant-based reconstruction (Figure 1), with 55.4% of surgeons utilizing implants for reconstruction over 80% of the time.

**Table 1:** Demographics of the plastic surgeon cohort, *N* = 329.

**Characteristics**	***N* (%)**
**WORK SETTING**	
University/teaching hospital	87 (26.44%)
Private practice	224 (68.08%)
Veterans Affairs (VA) hospital	0 (0%)
Other	14 (4.25%)
Missing	4 (1.21%)
**YEARS IN PRACTICE**	
<5	22 (6.75%)
5–9	47 (14.42%)
10–14	57 (17.48%)
15–19	52 (15.95%)
20–24	62 (19.02%)
25 or more	83 (25.46%)
Missing	3 (0.09%)
**GENDER**	
Male	266 (81.10%)
Female	62 (18.90%)
Missing	1 (0.03%)
**ROUTINE ATTENDANCE OF A BREAST MULTIDISCIPLINARY**	
**CONFERENCE**	
Yes	177 (54.80%)
No	131 (40.56%)
I do not have access to a breast multidisciplinary conference	15 (4.64)
Missing	6 (1.8%)
**PROPORTION OF PRACTICE DEVOTED TO BREAST RECONSTRUCTION**	
<20%	122 (37.31%)
20–50%	136 (41.59%)
51–80%	47 (14.37%)
>80%	22 (6.73%)
Missing	2 (0.06%)
**IN YOUR PRACTICE, WHAT PROPORTION OF PATIENTS RECEIVE**	
**IMPLANT BASED RECONSTRUCTION?**	
<20%	12 (3.69%)
20–50%	30 (9.23%)
51–80%	102 (31.38%)
>80%	179 (55.08%)
Unknown	2 (0.62%)
Missing	4 (1.21%)
**NUMBER OF CPM RECONSTRUCTIONS PERFORMED IN A MONTH**	
0	24 (7.36%)
1	82 (25.15%)
2–4	163 (50.00%)
5–6	28 (8.59%)
>6	29 (8.90%)
Missing	3 (0.09%)

Responders reported the discussion of CPM was most commonly initiated by the patient (*N* = 162, 51.1%) followed by the breast surgeon (*N* = 122, 38.5%). Only 23 (7.3%) of responders reported CPM discussions were commonly initiated by the plastic surgeon. According to responders, patients were most likely to choose CPM due to a perceived increased risk of contralateral cancer, to avoid further biopsy or imaging, and an actual increased risk of contralateral cancer (Table 2).

**Table 2:** Plastic surgeons' rating of patient motivations to undergo CPM.

	**Almost Always *N* (%)**	**Frequently**	**Occasionally *N* (%)**	**Rarely *N* (%)**	**Almost never/never**	**Total**
Actual increased risk of contralateral breast cancer	45 (14.4)	111 (35.5%)	106 (33.9)	48 (15.3)	3 (0.9)	313
Perceived increased risk of contralateral breast cancer	117 (36.9)	157 (49.5)	35 (11.0)	6 (1.9)	2 (0.6)	317
Cosmesis/symmetry	32 (10.2)	121 (38.8)	101 (32.4)	43 (13.8)	15 (4.8)	312
Avoid future imaging/biopsies	81 (25.9)	152 (48.6)	58 (18.5)	17 (5.4)	5 (1.6)	313
Mistrust of surveillance	19 (6.1)	95 (30.5)	102 (32.8)	72 (23.2)	23 (7.4)	311
Physician recommendation	32 (10.2)	89 (28.3)	130 (41.4)	56 (17.8)	7 (2.2)	314
Survival benefit	28 (9.0)	77 (24.8)	95 (30.7)	81 (26.1)	29 (9.4)	310
Lack of resources for adequate follow up/surveillance	2 (0.6)	12 (3.8)	34 (10.8)	118 (37.5)	149 (47.3)	315
Additional findings on imaging	9 (2.9)	56 (18.3)	140 (45.8)	81 (26.5)	20 (6.5)	306

### Physician Attitudes/Knowledge of CPM

A total of 325 surgeons responded to the question, “Have you ever felt uncomfortable with a patient's choice to proceed with a contralateral prophylactic mastectomy?” Of these, 60 (18.5%) reported discomfort. On univariate analysis, surgeons were more likely to feel uncomfortable with CPM if they did not routinely attend a multidisciplinary breast conference. Otherwise, there was no difference in comfort level by years in practice, amount or type of breast reconstruction performed, or hospital work setting (Table 3). Of the 60 surgeons reporting discomfort with CPM, 57 responded to the question, “Why have you ever felt uncomfortable with a patient's decision to undergo CPM?” The most common themes that emerged from the qualitative analysis were: concern regarding the increased risk of additional surgery in the setting of a limited oncologic benefit and a perceived lack of understanding by the patient of the anticipated outcome of CPM (Table 4). In addition, 21 surgeons surveyed responded to the question “Why have you ever felt uncomfortable with a patient's decision to undergo CPM?” with text articulating their reasons why they were comfortable with a patient's choice for CPM. The most common themes that emerged were the principle of respect for autonomy as the basis for comfort, followed by a recognition of non-oncologic benefits of CPM (Table 5).

**Table 3:** Plastic surgeon characteristics and CPM comfort level.

**Physician characteristics**		**Uncomfortable with patient's choice to proceed with CPM *N =* 60**	***p*-value**
	***N*** **(%)**	***N*** **(%)**
**GENDER**			
Male	266 (81.1)	45 (16.9)	0.25
Female	62 (18.9)	15 (24.2)
**ROUTINELY ATTEND BREAST CONFERENCE**			
Yes	177 (54.8)	23 (13)	0.023
No	146 (45.2)	37 (25.3)
**NO. OF YEARS IN PRACTICE**			
<10	69 (23)	10 (16.9)	0.53
10–19	109 (34)	19 (17.4)
20+	145 (43)	30 (20.7)
**WORK SETTING**			
University/teaching hospital	87 (26.8)	22 (25.3)	0.13
Private Practice	224 (68.9)	35 (15.6)
Non-teaching Hospital	14 (4.3)	2 (14.3)
**% OF PRACTICE BREAST RECONSTRUCTION**			
<20	122 (37.3)	24 (19.7)	0.46
20–50	136 (41.6)	20 (14.7)
51–80	47 (14.4)	10 (21.3)
>80	22 (6.7)	6 (27.3)
**PROPORTION IMPLANT BASED (%)**			
<20	12 (3.7)	3 (25)	0.76
20–50	30 (9.3)	7 (23.3)
51–80	102 (31.6)	17 (16.7)
>80	179 (55.4)	31 (17.3)
**CPM RECONSTRUCTION PER MONTH**			
0–1	106 (32.5)	18 (17)	0.27
2–4	163 (50)	35 (21.5)
5+	57 (17.5)	7 (12.3)

**Table 4:** Qualitative analysis of plastic surgeons' comfort level performing CPM.

**Broad category**	**Supporting elements**	**Representative quotations**
Risk/benefit ratio	Lack of oncologic benefit	“No survival benefit and a very normal non-cancer breast.”
		“Patients with a low risk of contralateral malignancy.”
	Loss of sexual function of breast	“They have 5–7% risk of (contralateral) cancer, and 100% chance of loss of sensation and sexuality from its loss. Also complication for bilateral goes up to 40%.”
		“Concern about the need to perform this from an oncologic point of view. Defeminization.”
	Risk of perioperative complication	“Increased surgery risks of infection, DVT, capsular contracture/pain. Harder recovery, possible delay of chemotherapy if any wound healing complications.”
		“Soft indications combined with increased complications with bilateral immediate reconstructions.”
Lack of understanding	Cosmetic expectations	“They often have the mistaken idea that sacrificing the other side is best for symmetry.”
	Overestimation of benefit of CPM	“I fear they are reacting to fear and media coverage. They are misled about the protective benefit.”
		“When I think they don't understand it makes no difference in survival and it increases risks.”

**Table 5:** Qualitative analysis of surgeon's comfort level in performing CPM, surgeons reporting no discomfort with a Patient's Request for CPM.

**Broad category**	**Supporting elements**	**Representative quotations**
Respect for Autonomy		“I think it's the patient's right to decide how to go about risk reduction, with proper guidance from breast oncologic and reconstructive surgeons.”
		“The breast belongs to the patient and it's her decision what to do with it to give her the best balance of peace of mind vs. deformity with subsequent reconstruction.”
Benefit of CPM	Decreased anxiety	“Most patients feel relieved at their decision to not worry anymore.”
		“I am generally in favor if it provides peace of mind.”
	Symmetrical reconstruction/ease of surveillance	“Less mammograms, easier symmetry, peace of mind for patient”

The majority of responders reported the 5-year risk of contralateral breast cancer in a patient without additional risk factors to be 5% or less (*N* = 202, 71.1%). Most responders stated there is little to no evidence that CPM effects disease specific survival (*N* = 264, 86.5%). When asked to estimate the overall complication rate of CPM compared to UM with all types of reconstruction, 41.6% (*N* = 129) reported no difference, 52.23%(*N* = 165) a 2-fold risk, 3.9% (*N* = 12) a 3-fold risk, and 1.3% (*N* = 4) a 4-fold risk (Table 6). Nevertheless, 63.1% (*N* = 197) of surgeons were of the opinion that the side effects of a second mastectomy were worth the benefit.

**Table 6:** Plastic surgeon's knowledge about CPM.

**Questions**	***N* (%)**
**What is your overall impression of the evidence to**	
**support a contralateral prophylactic mastectomy to**	
**prolong disease-specific survival? (*****N=*** **305)**	
Strong evidence	5 (1.6)
Moderate evidence	36 (11.8)
Limited to weak evidence	144 (47.2)
No evidence of survival benefit	120 (39.3)
**The risk of overall complications for patients undergoing**	
**CPM compared to unilateral mastectomy (with all types of**	
**Reconstruction) is: (*****N =*** **310)**	
There is no difference	129 (41.6)
Approximately two times the risk	165 (53.2)
Approximately three times the risk	12 (3.9)
Approximately four times the risk	4 (1.3)
Approximately five times the risk	0 (0.0)
**In a patient with invasive ductal carcinoma and no**	
**additional risk factors, what risk do you quote of**	
**developing a contralateral breast cancer over**	
**a 5-Year Period? (*****N =*** **284)**	
<2%	93 (32.8)
2–5%	109 (38.4)
6–10%	37 (13.0)
11–15%	26 (9.2)
16–20%	14 (4.9)
>20%	5 (1.7)

## Discussion

To our knowledge, this is the first survey study of PS perspectives of CPM. According to PS surveyed, discussion of CPM is patient driven and dominated by a perceived increased risk of contralateral cancer. Most PS demonstrated knowledge of the expected oncologic benefit and increased complications risk of CPM consistent with current data. However, most responders believed the risk to be worth the benefit. Only 18.5% of responders reported discomfort with a patient's decision to undergo CPM. PS discomfort was due to the limited oncologic benefit, increased operative risk, and concern for patients overestimating the benefit of CPM. Surgeon comfort was based on the principle of respect for autonomy and non-oncologic benefits of CPM.

A survey of the American Society of Breast Surgeons (ASBrS) assessing perspectives of CPM from the breast surgeon's perspective was reported in 2016 (19). Similar to this survey, respondents reported that patients most often introduce the topic of CPM and PS introduce CPM infrequently. Respondents of both surveys reported a perceived increased risk of contralateral cancer as the most common reason patients request CPM. In contrast to our survey, 55.6% of ASBrS responders reported discomfort with a patient's choice for CPM, although reasons for discomfort were similar: overtreatment, an unfavorable risk benefit ratio, and lack of patient understanding of the expected benefit. ASBrS members similarly cited respect for autonomy a primary reason for comfort with a patient's choice for CPM (20). The disparity of comfort level between breast surgeons and PS may be due to their different roles, with PS carrying the primary responsibility for cosmetic rather than oncologic outcome. From the open-ended responses on PS comfort, responders placed a value in the non-oncologic benefits of CPM such as peace of mind and symmetry. Differences between ASBrS member and PS responses as well as increased discomfort in PS who do not attend multidisciplinary breast conference, suggest that greater PS collaboration through modalities such as interdisciplinary conferences may be helpful in improving the decision making process with CPM.

The role of CPM in enhancing the peace of mind for breast cancer patients is a complex topic. Few studies have specifically looked at post-operative anxiety levels. In a prospective observational study of mastectomy patients, Momoh et al. reported at 1 year postoperatively, anxiety levels did not differ between patients undergoing UM vs. CPM (21). Conversely, in the Young Woman's Breast Cancer Study (YWS), receipt of CPM vs. UM or breast conserving surgery was associated with less fear of recurrence (7). Data on overall satisfaction with CPM are more robust, showing that women report high rates of satisfaction with their decision for CPM. In a survey of women undergoing CPM, 79% of women who had CPM and reconstruction reported being satisfied with CPM > 10 years after the procedure and 92% of women reported they would choose CPM again (22). In the YWS of CPM patients, 94% of whom underwent reconstruction, 80% reported that they were extremely confident with their decision 90% reported they would definitely choose CPM again (23). Many women in the YWS also reported outcomes that were worse than they expected including the number of surgeries (33%), parasthesias (28%), and a worse sense of sexuality (42%). Other quality of life parameters appear to be similar between UM and CPM patients. In a survey of over 7,500 women who underwent CPM or UM, Hwang et al. reported the results of a validated quality of life survey (BREAST-Q) and found on multivariable analysis that women who had undergone CPM reported higher breast satisfaction. However, this difference was so small it was unlikely to be clinically significant. In addition, there was no difference in other quality of life domains between the two groups, including physical and sexual well-being (24). Whether or not CPM achieves peace of mind and positively affects quality of life in breast cancer patients continues to be an area of debate (25–27). Invariably, this answer ultimately rests in the individual medical and non-medical values and goals of the breast cancer patient.

Many patients also consider cosmetic outcomes when choosing CPM. In the YWS survey study, 57% of respondents reported that a desire for symmetry was a very important factor. Only 15% reported symmetry was not important at all (23). The role of CPM in attaining symmetry in breast reconstruction may be affected by the type of reconstruction available. Matching the healthy breast can be easier with autologous reconstruction as it more closely resembles the native breast tissue. Over time, the asymmetry with a unilateral implant reconstruction is more noticeable as the patient's native breast changes shape with ptosis or with changes in body weight. In this cohort of surgeons, the majority reported performing implant-based reconstruction >80% of the time, which likely affected reported perceptions of CPM. In a prospective observational study of women undergoing CPM or UM for breast cancer with reconstruction, patient-reported satisfaction was significantly better in women undergoing CPM vs. UM with implant reconstruction, with no difference in satisfaction levels between UM and CPM in patients undergoing autologous reconstruction (21). In a review of the Surveillance, Epidemiology and End Results database (SEER) from 2000 to 2010, patients with implant reconstruction were more likely to undergo CPM compared to patients undergoing autologous reconstruction (28). Oncoplastic procedures such as breast reduction, breast implantation and mastopexy can be performed to match the breasts. However, these procedures may be underutilized. In a review of the American College of Surgeons National Surgical Quality Improve Program (NSQIP), of the 24,191 women undergoing mastectomy with immediate reconstruction between 2005 and 2012, only 3.7% underwent matching procedures. Conversely, in a NSQIP analysis of 20,501 patients undergoing mastectomy for a unilateral breast cancer between 2005 and 2013, 35.3% underwent CPM (29). While these two groups are not directly comparable, the discrepancy between the numbers of oncoplastic procedures and CPM in these two series is striking. We did not query PS on their practice of balancing procedures for breast cancer and therefore cannot report on this use of oncoplasty as a cosmetic alternative to CPM in this group.

In this survey, the majority of PS exhibited knowledge of contralateral cancer rates, survival expectations, and complication rates of CPM consistent with published literature. Those PS who reported discomfort with CPM cited the limited oncologic benefit and increased complication rate as a common reason for discomfort. The incidence of a contralateral breast cancer in a patient without a strong family history or deleterious genetic mutation is cumulative, but low, ranging from 0.3 to 1% per year depending on the specific patient (30, 31). There are no randomized trials assessing survival with CPM. Data supporting a survival benefit is retrospective and subject to selection bias and confounding factors such as patient disease and tumor characteristics (32–34). The post-operative complication rate has been shown to be higher with CPM and reconstruction compared to UM with reconstruction, although some data show similar complication rates. Osman et al reported that patients experienced wound complications in 5.8% of CPM cases vs. 2.9% of UM cases (35). A single institution study found that CPM patients were 1.5 times more likely to experience complications than UM patients and 2.7 times more likely to experience a major complication (36). A more recent study comparing CPM to UM described longer hospital stays and higher transfusion rates for CPM patients than UM patients, but similar rates for site infection, prosthesis failure, and medical complications were observed (29).

This study has several limitations. The response rate was only 12.5%, although this is en par with other physician surveys through the ASPS (37–39). The survey was only distributed twice and responders were not compensated. The demographic data of ASPS is comparable with the responders of this survey in terms of practice setting and years in practice (49, Appendix 2). The majority of PS did not report discomfort with CPM and we did not specifically ask why PS were comfortable with the procedure. Qualitative data on PS comfort was only available from responders who discussed reasons for comfort in the free text responses solicited from a question about discomfort. If we had solicited free text from a question specifically asking about surgeon comfort, we may have elicited more responses outlining why PS may view CPM as a valuable procedure.

## Conclusion

This study demonstrates that the majority of PS report knowledge of expected oncologic and complication rate outcomes consistent with the literature. PS recognize the limited or lack of data for survival benefit and the low risk of contralateral breast cancer in most patients. Despite this, PS perceive CPM as worth the additional surgery compared to a UM, suggesting PS appreciate a non-oncologic value of CPM. Most PS report comfort with CPM due to a respect for autonomy and appreciation of the cosmetic benefits of a symmetrical reconstruction. PS also recognize quality of life benefits such as decreased surveillance and peace of mind. Given the prominent role that reconstructive surgery can play in the CPM decision making process, further research is needed to elucidate how the different reconstructive options can influence patients' decisions for CPM. In addition, as guidelines for CPM continue to be established and revised, PS could be used as a resource to provide input on the cosmetic aspects of CPM.

## Author Contributions

EB is responsible of concept design and writing of paper. CL contributed to writing the paper. CH contributed to writing of the paper and statistical analysis. RB-L contributed to concept design. SS contributed to concept design.

### Conflict of Interest Statement

The authors declare that the research was conducted in the absence of any commercial or financial relationships that could be construed as a potential conflict of interest.
