# Supplementary material for: Plastic and Reconstructive Surgeons' Knowledge and Comfort of Contralateral Prophylactic Mastectomy: A Survey of the American Society of Plastic Surgeons
Source: Front Oncol. 2019 Jan 9;8:647. doi: 10.3389/fonc.2018.00647 (PMC6334534; doi:10.3389/fonc.2018.00647)
Supplement: Supplementary file 1 [file Data_Sheet_1.PDF]

1. Appendix 1. Contralateral Prophylactic Mastectomy Survey

1. Which one of the following best describes your work setting?

- ◯ University/teaching hospital (includes all settings with teaching medical students or residents)
- ◯ Private practice-nonteaching
- ◯ Hospital employed-nonteaching
- ◯ Veterans Affairs (VA)

2. How many years have you been in practice?

____

3. What proportion of your practice is devoted to breast reconstruction?

- ◯ <20%
- ◯ 20-50%
- ◯ 50-80%
- ◯ > 80%

4. Please indicate your gender.

- ◯ Male
- ◯ Female

5. Do you routinely attend a breast multidisciplinary conference?

- ◯ Yes
- ◯ No
- ◯ N/A I do not have access to a breast multidisciplinary conference

6. In your practice, what proportion of patients receive IMPLANT based reconstruction?

- ◯ < 20%
- ◯ 20-50%
- ◯ 50-80%
- ◯ > 80%

7. Please estimate the number of reconstructions you perform for contralateral prophylactic mastectomy in a month?

- 0
- 1
- 2-4
- 4-6
- >6

8. Have you ever felt uncomfortable with a patient's choice to proceed with a contralateral prophylactic mastectomy?

- No
- Yes

9. Why have you felt uncomfortable with a patient's decision to undergo a contralateral prophylactic mastectomy?

10. In discussing the surgical management of breast cancer, contralateral prophylactic mastectomy is most commonly initiated by

- Breast Surgeon
- Plastic surgeon
- Patient
- Other physician
- Patient family/friends

11. Please rate your patients' motivations for choosing contralateral prophylactic mastectomy

	Almost always	Frequently	Occasionally	Rarely	Almost never/never
Actual increased risk of contralateral cancer					
Perceived increased risk of contralateral cancer					
Cosmesis/symmetry					
Avoid future imaging/biopsies					
Mistrust of surveillance					
Physician recommendation					
Survival benefit					
Lack of resources for adequate follow up/surveillance					
Additional findings on imaging					

Other (please specify)

12. Please rate your agreement with the following statement:

In patients with NO additional risk factors for contralateral breast cancer, the side effects of a second mastectomy are generally not worth the benefits.

- Strongly DISAGREE
- Somewhat DISAGREE
- Neutral
- Somewhat AGREE
- Strongly AGREE

13. The risk of overall complications for patients undergoing CPM compared to unilateral mastectomy (with all types of reconstruction) is:

- There is no difference
- Approximately two times the risk
- Approximately three times the risk
- Approximately four times the risk
- Approximately five times the risk

14. In a patient with invasive ductal carcinoma and no additional risk factors , what risk do you quote of developing a contralateral breast cancer over a 5-year period?

- < 2 %
- 2-5 %
- 5-10%
- 10-15%
- 15-20%
- > 20%

15. What is your overall impression of the evidence to support contralateral prophylactic mastectomy to prolong disease-specific survival?

- No evidence of survival benefit
- Limited to weak evidence of survival benefit
- Moderate evidence of survival benefit
- Strong evidence of survival benefit
