# Supplementary material for: Plastic and Reconstructive Surgeons' Knowledge and Comfort of Contralateral Prophylactic Mastectomy: A Survey of the American Society of Plastic Surgeons
Source: Front Oncol. 2019 Jan 9;8:647. doi: 10.3389/fonc.2018.00647 (PMC6334534; doi:10.3389/fonc.2018.00647)
Supplement: Supplementary file 2 [file Data_Sheet_2.PDF]

Appendix 2. ASPS Membership Demographics

Type of Practice	ASPS %
Solo	52.1%
Solo practice-shared facility	6.1%
Small plastic surgery group practice (2-5 plastic surgeons)	18.4%
Large plastic surgery group practice (6 or more plastic surgeons)	2.6%
Medium multi-specialty group practice (6-20 physicians)	1.5%
Large multi-specialty group practice (more than 20 physicians)	6.5%
Academic	11.9%
Military	0.9%

Gender	ASPS %
Male	84%
Female	16%

Years in practice distribution	ASPS %
Less than 5	14.1%
5 - 9	16.2%
10 - 14	14.1%
15 - 19	17.2%
20 - 24	15.0%
25 years or more	23.5%

Age distribution	ASPS %
Under 35	2.2%
35 - 44	23.1%
45 - 54	29.0%
55 - 64	29.2%
65 and over	16.5%

Regional distribution	ASPS %
South Atlantic	21.6%
Pacific	18.7%
Middle Atlantic	15.2%
East North Central	12.0%
West South Central	11.0%
Mountain	6.7%
West North Central	5.2%
New England	4.8%
East South Central	4.8
